# Beef Cattle Preference and Usage of Environmental Enrichments Provided Simultaneously in a Pasture-Based Environment

**DOI:** 10.3390/ani12243544

**Published:** 2022-12-15

**Authors:** Emily J. Dickson, Dana L. M. Campbell, Caroline Lee, Jim M. Lea, Paul G. McDonald, Jessica E. Monk

**Affiliations:** 1 Agriculture and Food, Commonwealth Scientific and Industrial Research Organisation (CSIRO), Armidale, NSW 2350, Australia; 2 School of Environmental and Rural Science, University of New England, Armidale, NSW 2351, Australia

**Keywords:** environmental enrichment, beef cattle, extensive production, welfare, behaviour

## Abstract

**Simple Summary:**

Environmental enrichment can improve livestock welfare by increasing environmental complexity to promote a greater range of natural behaviours. However, there is limited understanding of the need for and impacts of enrichments for extensively managed beef cattle, which are sometimes kept in grassed paddocks with no other features. This trial assessed which enrichments beef cattle preferred and utilised in a barren paddock environment. Eight groups of seven Angus steers housed on pastured paddocks devoid of natural or artificial features were observed during daylight hours over a period of three weeks after being presented with four enrichments simultaneously: a cattle brush, piece of hanging rope, a tree stump, and a woodchip pile. The brush, stump, and woodchip maintained a higher level of use than the rope, based on the number of interactions and the amount of competition over the enrichments, although enrichment use generally decreased over time. The inclusion of these enrichments can increase the complexity of barren pasture environments and allow for increased expression of natural behaviours, potentially contributing to improved welfare.

**Abstract:**

Environmental enrichment can improve livestock welfare through increasing environmental complexity to promote a greater range of natural behaviours. However, there is limited understanding of the need for and impacts of enrichments for extensively managed beef cattle that can sometimes be kept in grassed paddocks devoid of additional natural and artificial features, i.e., ‘barren pastures’. This trial assessed which enrichments beef cattle preferred and utilised in a barren paddock environment. Eight groups of seven Angus steers housed on pastured paddocks devoid of natural or artificial features were observed during daylight hours for two days a week over a period of three weeks, after being presented with four enrichments simultaneously: a cattle brush, a piece of hanging rope, a tree stump, and a woodchip pile. Although enrichment use generally decreased over time, the brush, stump, and woodchip maintained a higher level of use than the rope, based on the frequency of interactions and number of displacements around the enrichments (both *p* < 0.001). This suggests that the brush, stump, and woodchip pile were more valuable resources to the cattle, allowing for grooming and lying behaviours, although oral manipulations also occurred on the stump, woodchip, and rope. The inclusion of these enrichments can increase the complexity of barren pasture environments and allow for the increased expression of natural behaviours, potentially contributing to improved welfare.

## 1. Introduction

The Australian beef industry is the most common and geographically dispersed agricultural activity in the country, with around 50% of all agricultural farms carrying beef cattle [1]. Environmental features can vary greatly between regions, farms, and paddocks through differing climates, terrain, water sources, pasture composition, and the presence of other natural or artificial objects. This variation in surroundings likely results in differing degrees of environmental enrichment opportunities for pasture-based cattle, ranging from environmentally complex areas through to ‘barren’ pastures (i.e., devoid of natural or artificial objects beyond grassed fields). There has been a large amount of research into the use and benefits of environmental enrichment provision for dairy cattle welfare and production [2], as well as enrichment provision for beef cattle in feedlots [3,4]. However, little is known about whether a barren pastured environment will impact beef cattle behaviour and welfare, and if there is utilisation of and preference for enrichments when provided to cattle kept in these conditions.

One major goal of environmental enrichment is to increase the range of normal, species-specific behavioural expression [5]. Grazing, ruminating, and resting/lying occupy over 90–95% of beef cattle’s time budget when housed on varying types of pastured environments. Other behaviours that occupy the remaining 5–10% of their time budget include social behaviours, drinking, excreting, walking, and investigating or interacting with an object within the paddock [6]. The degree of environmental complexity under which all these observations have been conducted is not certain, but common behaviours of grazing, resting, then walking in beef cattle across paddocks with some amount of vegetation have been reported [7]. Studies on feedlot and dairy cattle can give some indication of preferred enrichments such as mechanical brushes [8,9], other grooming devices [4], and hanging ropes [9,10]. However, these animals are generally housed with either no or restricted access to pasture [2], which impacts their time budgets and may allow more time for enrichment use in place of grazing time. Barren pasture environments do allow for grazing, walking, and resting but may still be restrictive of a full cattle behavioural repertoire and could be optimised by enrichment provision. Lying time may be increased through the provision of alternative surface substrates, while additional behaviours that may be facilitated through enrichment for pasture-based beef cattle could include grooming and oral behaviours.

Grooming is seen in cattle at relatively low rates, at approximately 2% of the day, and includes self-grooming, allogrooming, and grooming on objects [7]. It is thought to have multifaceted causes, including satisfying a physical requirement to stay clean through the removal of dirt, ectoparasites, and other substances, and also potentially as a mechanism to allow for de-arousal [11]. Self-grooming and allogrooming were not impacted when pasture-based beef cattle were allowed access to trees for grooming [12]; however, self-grooming, but not allogrooming, were increased when dairy calves were provided with a rotating brush [13]. Therefore, a barren paddock with no trees or similar objects may deprive cattle of the opportunity to perform some grooming behaviours, which may not be substituted with self-grooming and allogrooming.

Oral manipulation of objects, particularly chewing, licking, and nibbling, are also seen in cattle, notably dairy calves and intensively managed cattle [9,10,14]. When cattle are in a feedlot environment or on thick improved pastures (i.e., grazing lands not used for cropping), eating time is reduced and replaced with other oral behaviours when compared to cattle in paddocks with low pasture availability [15]. Although some oral behaviours such as tongue rolling may be defined as stereotypic behaviours [16], other exploratory behaviours may be rewarding to the cattle. Beneficial oral behaviours could be encouraged in both paddock-based and intensive systems through the provision of objects such as ropes, which can be chewed or suckled.

Lying is an important behavioural requirement of beef cattle, who have been observed to spend around 11 to 12 h a day lying at pasture [17,18]. Lying is often promoted for indoor-housed dairy cattle through the provision of bedding materials such as straw, wood shavings and chips, or sand [19]. However, lying behaviour shows improvements when dairy cattle have access to pasture [20,21], dependent on pasture quality and grazing requirements [22]. When provided with or without a woodchip area at pasture, lactating dairy cattle did not show consistent differences in lying time and most lying was on the grass [23]. However, it is unknown if beef cattle would use additional bedding in barren pastures, or whether this resource would enhance time spent on this activity through either increased lying time and/or bouts, particularly if inclement weather resulted in muddy surfaces [24,25].

Overall, environmental enrichment has the potential to improve welfare by reducing frustration and boredom felt when an animal is housed in a barren or inappropriate environment and is unable to perform specific behaviours [5,26,27]. Additionally, there is increasing interest in enrichment as a method to promote positive experiences. For example, grooming in cattle has been suggested to be a hedonistic behaviour, and is linked to positive affective states [28,29,30].

The aim of the current study was to determine the preferred environmental enrichments of small groups of beef cattle housed in paddocks barren except for pasture, water, and the chosen enrichments; a rope, brush, stump, and woodchip pile. It was predicted that, over a period of 3 weeks, enrichment use would peak during the first week due to novelty, while the use of any highly valued enrichments would be maintained over time. In addition, high-value enrichments were expected to result in a greater number of displacements, as an indication of increased competition over the resource. Finally, the rope was expected to be least valued by the cattle, as comparable oral behaviours could potentially be displayed through grazing, whereas no comparable substitutes for the brush, stump, and woodchip pile would be available in the pasture.

## 2. Materials and Methods

### 2.1. Ethical Statement

The procedure of this study was approved by the CSIRO Agriculture Animal Ethics Committee (Armidale, ARA 21-09), under the New South Wales Animal Research Act 1985.

### 2.2. Facilities and Animals

The experiment was undertaken at CSIRO, FD McMaster Laboratory, Chiswick, Armidale, NSW from October to December 2021. Weather data were collected from an onsite MEA weather station (Green Brain, 41 Vine Street, Magill, SA, Australia). The average 24 h daily temperature was 14.3 °C (±0.4 °C), average daily rainfall was 4.0 mL (±1.0 mL, average humidity was 80.6% (±1.4%), and average wind speed was 13.2 km/h (±0.8 km/h). It was noticed that some areas of the paddocks became waterlogged and muddy due to rainfall, but this was not quantified.

**Figure 1:**
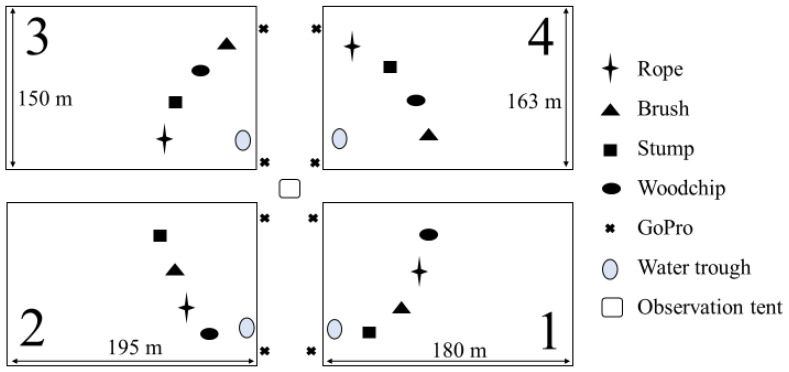
Schematic of the 4 experimental paddocks (1–4) showing sizes and location of enrichments (rope, brush, stump, woodchip pile), water troughs, GoPro cameras, and observation tent. Paddocks 1 and 4 were the same dimensions (160 m × 180 m), and paddocks 2 and 3 were the same dimensions (150 m × 195 m). Not drawn precisely to scale.

**Figure 2:**
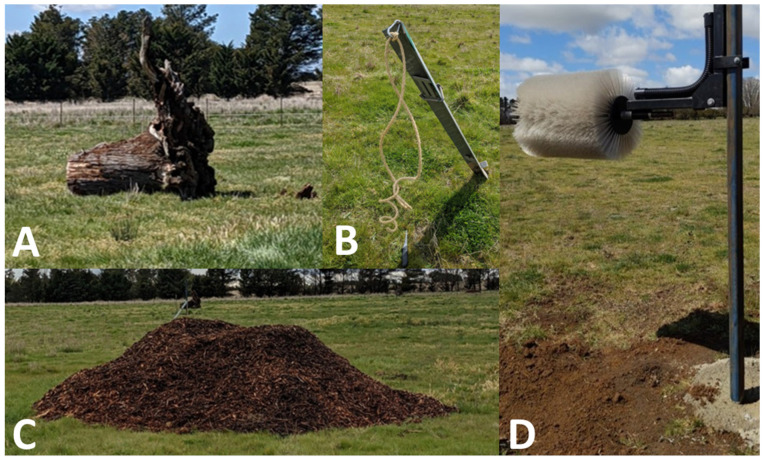
Photographs of the enrichments used; tree stump (**A**), hanging rope (**B**), woodchip pile (**C**), and cattle brush (**D**).

Four neighbouring paddocks approximately 3 hectares each in size were used. Each contained a water trough and four enrichments, which were positioned 30–35 m apart in proximity of the observation area for both live data collection and video recording (Figure 1), all of which were characterised by a similar mix of native and introduced pasture. Pasture data were not collected for this study, although experienced staff deemed the quantity sufficient for the number of animals grazing without any requirement to provide additional feed. No other objects, such as trees, were in the paddocks, and electric fences were used to prevent the use of fences for grooming. The four enrichment objects (Figure 2) included were:

- Cattle brush (Redpath, 16 Bounty Place, Kelvin Gove, Palmerston North 4414, New Zealand)
- Tree stump placed on side with semi-intact root balls (found at the Chiswick site, approximately 1.6 m × 2.4 m × 1.5–2.5 m)
- Sisal rope hanging from a slanted metal post, knotted at ends (10 mm diameter, two strands 1 m in length)
- Woodchip mound (3 m in diameter, 1–1.5 m high)

Fifty-six Angus steers of approximately one year of age kept in groups of seven animals each across two cohorts were used in the study. Cattle from Cohort 1 were sourced directly from the Chiswick site, whilst Cohort 2 were purchased from a commercial producer, and arrived on site 3–4 weeks before they entered the testing paddocks. Prior to the current study, all cattle were housed in a range of standard commercial paddocks and were expected to have experience with objects such as trees and stumps, but were naïve to enrichments such as the brush, rope, and woodchip.

Cattle were weighed using walk-over weighing scales in a crush 1–2 weeks prior to testing commencing, and groups within cohorts were balanced for weight (average weight ± SE Cohort 1 = 303 ± 5 kg; Cohort 2 = 333 ± 6 kg). All cattle were retained on the Chiswick farm at completion of the study. 

### 2.3. Study Design and Data Collection

Prior to cattle entering their enriched paddocks, individuals within each group were marked with numbers 1–7 using livestock paint (Leader Products Pty Ltd., Craigieburn, VIC, Australia). This was repainted once per week on a non-observation day, to ensure ease of reading. Cattle were walked into the centre of their test paddock and had free access to enrichments within the paddock for a period of up to 3 weeks. Group placement into the paddocks was staggered to facilitate ease of behavioural observations, with Day 1 considered as the day each group was introduced to their respective test paddock. Initially, Group 1 was continuously observed for 12 h (07:00 to 19:00) for four consecutive days to determine peak enrichment use times and refine the behavioural ethogram. After that, Groups 2–4 were placed into each paddock over 3 consecutive days, where they remained for up to 3 weeks. Observations were made on Days 1, 2, 8, 9, 15, and 16. The entirety of Cohort 1 completed their time in the test paddock before Cohort 2 was introduced to the paddocks. 

On each observation day, the cattle were watched between 07:00 and 19:00 across Cohort 1 and from 06:30 to 19:45 across Cohort 2 due to lengthening daylight hours. Preliminary findings in Cohort 1 indicated the mornings and evenings to be the periods of peak enrichment use. All groups were observed live on their first two days, then a combination of live and video recordings was utilised for behavioural data collection on subsequent days (live from 15:00 to 19:00 for Cohort 1, and 16:00 to 19:45 for Cohort 2, with video recordings the remainder of time). Group 1 was an exception, with only video recordings used for days 9 and 16.

Live observations of behaviours were recorded by a minimum of two personnel at a time through an annotation application (CSIRO AnnoLOG v 1.0.23, St. Lucia, Brisbane, QLD, Australia) installed on a Samsung Galaxy Tab A 7.0 (Samsung, Seoul, Korea). All personnel were trained by a single individual (ED) who devised the behavioural ethogram (Table 1). The time a relevant behaviour began, the duration, and the animal ID were recorded. Video recordings were taken on GoPros (HERO 7 and 8, San Mateo, CA, USA), set up in locations as per Figure 1. Videos were replayed on “VLC media player” and time of relevant behaviours, duration, and Animal ID (when visible) were recorded.

Frequency of enrichment use was determined by counting the number of bouts. A bout ended when an animal moved over a body-length away from an enrichment, or had no interaction with an enrichment for 30 s or more. 

### 2.4. Data and Statistical Analyses

Data from the AnnoLOG application were directly imported into Excel and combined with data from video recordings. All data analyses were performed in “R” [31]. Main effects were considered significant at *p* ≤ 0.05 and trends at 0.05 < *p* ≤ 0.10. 

#### 2.4.1. Latency to Interact with Enrichments

The latency to first interact with an enrichment was obtained from the Day 1 data for all animals. This was analysed using the “survival” package [32], with a stratified Cox proportional hazards model. An animal that failed to interact with a specific enrichment within 11 h was deemed a censored result. Each individual was treated as an experimental unit. To account for the effect of ‘Group’, it was fitted as a random effect.

#### 2.4.2. Daily Enrichment Use

Summary statistics were calculated to compare the raw durations spent performing specific behaviours with enrichments, as a percentage of total time spent interacting with enrichments, over all groups (*n* = 8) and observation days (*n* = 6).

To examine any potential “queuing” effect for a specific enrichment, we compared the total duration interacting with an enrichment to the time spent “Near” (within one body-length of an enrichment with no other interaction).

Daily data were then separated into four Early/Late AM/PM time-period categories. Observation periods were each 3 h for Cohort 1, and 3–3.75 h for Cohort 2, to account for different hours of observation between Cohorts 1 and 2 (Table A1). Due to technical issues with cameras resulting in a failure to record useable data for short periods, four time periods were excluded from the analysis (Group 1, Day 9, Early PM; Group 3, Day 9, Early AM; Group 5, Day 8, Late AM; Group 6, Day 9, Early PM).

The frequency of interactions with enrichments, displacements around enrichments, and daily weather data (temperature, rainfall, humidity, and wind speed) were then summarised by day number (*n* = 6), group (*n* = 8), enrichment (*n* = 4), and time-period category (*n* = 4). This resulted in a sample size of 752 unique events.

A principal components analysis (PCA) was performed using the “psych” package [33], to reduce the dimensionality of correlated weather variables. The first two principal components (PC) had eigenvalues greater than 1 and were retained for use in later models, with their loadings compared to raw data used to infer relationships between extracted PCs and real-world weather conditions. 

Models with all two-way interactions (Enrichment × Day, Enrichment × Time Period, and Enrichment × PCs) did not converge, and examination of raw data did not show obvious patterns, so these interactions were dropped to simplify models.

To analyse data examining enrichment use, the “lme4” package [34] was used. Group was the experimental unit. A two-stage modelling approach was taken, as there were many time periods in which no interaction occurred for specific enrichments. Firstly, a model was fitted for the probability that a specific enrichment was used or not, and then, only in time periods where use had occurred, a model was fitted for the frequency of use and number of displacements.

For modelling the probability of an enrichment being used, ‘Use’ was considered as a binary outcome (i.e., whether or not an enrichment was used within the time period by any animal), and a generalised linear mixed model (GLMM) was fitted of the form:

(1) $$\log_{e}(\frac{\pi_{i}^{\left(\mathrm{Use}\right)}}{1-{\;\pi }_{i}^{\left(\mathrm{Use}\right)}})={\mathrm{Enrichment}}_{\left(\mathrm{Brush},\;\mathrm{Rope},\;\mathrm{Stump},\;\mathrm{Woodchip}\right)}+{\mathrm{Day}}_{\left(1,\;2,\;8,\;9,\;15,\;16\right)}+{\mathrm{TimePeriod}}_{\left(\mathrm{EarlyAM},\;\mathrm{LateAM},\;\mathrm{EarlyPM},\;\mathrm{LatePM}\right)}+\;\mathrm{PC}1+\mathrm{PC}2+{\mathrm{Group}}_{\left(1,\;2\;\ldots \;8\right)}$$

where $\pi_{i}^{\left(\mathrm{Use}\right)}$ is the probability of an enrichment being used within a time period (i.e., P(Use_t_ = 1). ‘Group’ is a random effect, and all other effects are fixed. Model residuals were checked for normality and homoscedasticity using a visual assessment of Q-Q and residuals relative to fitted values plots. Significance testing of fixed effects was conducted using Wald Chi-square tests from the “car” package [35], and post hoc pairwise comparisons were made using contrast statements from the “emmeans” package [36].

For the second stages of analysis, a Poisson GLMM was fitted for the frequency of enrichment use, and a negative binomial GLMM for displacement data, using the “MASS” package [37], both generated using a binomial family link function. Model residuals were used as above to confirm adequate model fit, as well as significance testing of fixed effects and post hoc comparisons.

## 3. Results

### 3.1. Latency to Interact with Enrichments

**Figure 3:**
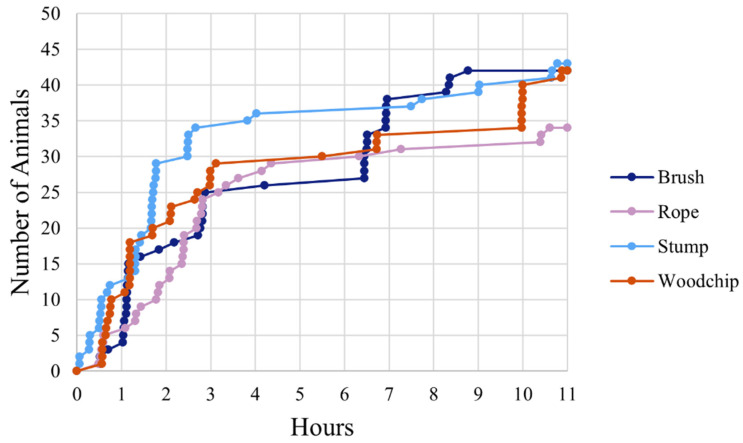
Kaplan–Meier curves indicating the latency for cattle to interact with different enrichments (brush, rope, stump, woodchip pile) during the first 11 h on Day 1 across 8 cattle groups (total animals = 56). Each point represents an individual’s first interaction with an enrichment.

Cattle approached the stump faster relative to all other enrichments, and fewer animals interacted with the rope in the first 11 h on Day 1 than with any other enrichment (Table 2, Figure 3). There was a trend (χ^2^ (3, *N* = 56) = 6.52, *p* = 0.09) for the animals to take longer to first interact with the woodchip pile than the stump (Table 2, Figure 3).

### 3.2. Daily Enrichment Use

**Figure 4:**
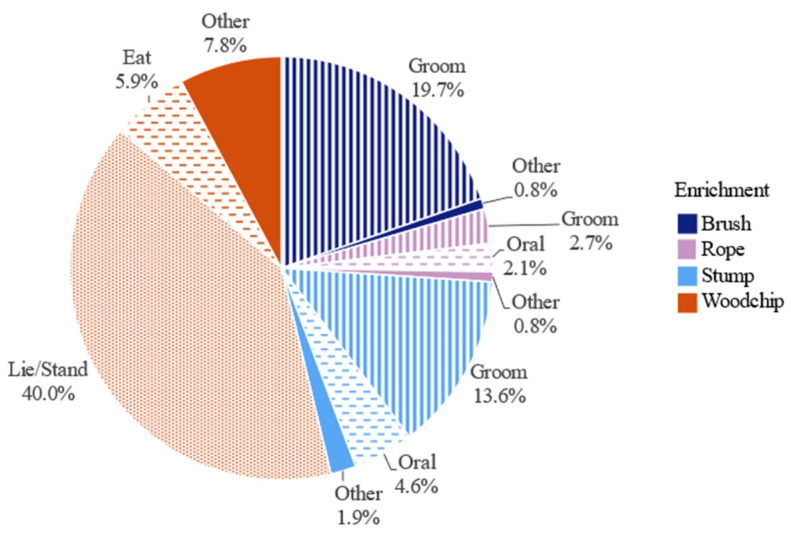
Duration spent performing specific behaviours on different enrichments, as a percentage of total time spent interacting with enrichments, over all groups (n = 8) and observation days (n = 6) (total = 182 h).

Across all groups and days, individual cattle were observed interacting with enrichments for a total of 182 h, accounting for approximately 4.4% of total observation time. From this, 53.7% of the total interaction time involved the woodchip pile, whilst 36.0% of interaction time involved grooming behaviours on the brush, rope, and stump, and a further 12.6% involved oral behaviours on the rope, stump, and woodchip (Figure 4). Cohort 1 was also observed performing 20 times more “Eat” behaviours on the woodchip than Cohort 2, whilst Cohort 2 performed 6 times more “Oral” behaviours on the stump than Cohort 1. Additionally, for every 60 seconds spent interacting with the brush and the stump, cattle would spend 65 seconds ‘Near’ but not interacting with these enrichments, and similarly 49 seconds near the rope and 15 seconds near the woodchip pile.

### 3.3. Principal Component Analysis 

**Figure A1:**
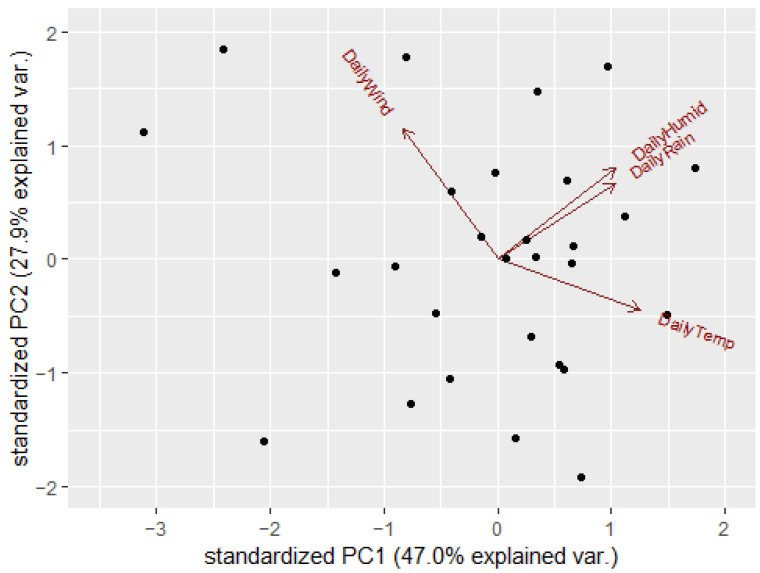
PCA vector graph for daily weather variables taken over each observation day of the study.

PC1 and PC2 both had eigenvalues > 1 (1.88 and 1.12, respectively), explaining 47.0% and 27.9% of weather variability, respectively, and were thus included in later models. Low PC1 values represented weather that was cool, dry, and windy, while higher values indicated hot, wet, and low wind conditions. PC2 was dry and low wind for low values, but windy and wet at high values (Table A2, Figure A1). 

### 3.4. Probability of Enrichment Use

Enrichment type significantly influenced the likelihood that it would be used, with cattle more likely to interact at least once with the brush than with the stump or woodchip pile during an individual time period (Table 3). The probability of an enrichment being used significantly decreased with day number and was also influenced by time period, with interactions more likely to occur in the late afternoon (Table 3). Weather did not have an impact on the probability of an enrichment being used at least once (Table 3).

### 3.5. Frequency of Interactions with Enrichments

**Figure 5:**
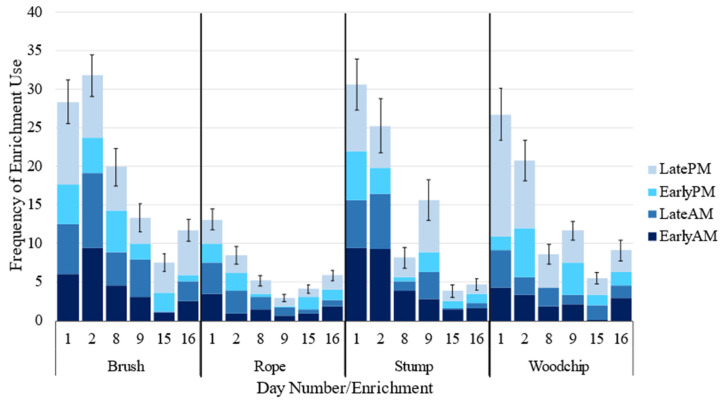
Mean frequency of enrichment interactions occurring at each enrichment, averaged across eight groups of seven cattle (±standard error for the day).

Given that an enrichment was used at least once during a time period, enrichment type influenced the frequency of enrichment use, with cattle interacting more frequently with the brush than the rope, but not the stump or woodchip pile (Table 4). Frequency of use decreased as day number increased, and was influenced by time period, with more interactions occurring in the morning than afternoon (Table 4). These patterns were also true when time periods where no interactions occurred were included (Figure 5). Weather also influenced frequency of use, with increasing rainfall and humidity resulting in decreasing use (Table 4).

### 3.6. Number of Displacements around Enrichments

**Figure 6:**
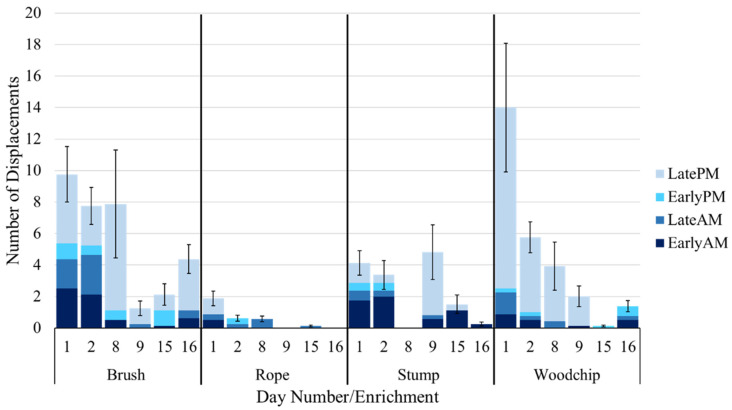
Mean number of daily displacements occurring at each enrichment, averaged across eight groups of seven cattle (±standard error for the day).

Given that an enrichment was used at least once during a time period, enrichment type influenced the number of displacements around enrichments, with the fewest displacements occurring around the rope, but no significant difference between the other enrichments (Table 5). The number of displacements decreased with day number and were also influenced by time period, tending to be more frequent in the late afternoon (Table 5). These patterns were also seen when time periods where enrichments were not used were included in daily averages (Figure 6). There was a tendency for hot or wet weather, and low wind conditions, to decrease number of displacements (Table 5).

## 4. Discussion

The types of enrichments that beef cattle will utilise when housed in pastures devoid of natural or artificial objects beyond grassed fields are relatively unknown. Identification of cattle enrichment preferences will provide the first step in ultimately determining their value and potential positive impacts on animal welfare and production. The current study aimed to determine beef cattle preferences for a variety of enrichments presented simultaneously in a pasture environment. Cattle interacted with all provided enrichments and, as predicted, there was a general pattern of enrichment use decreasing over time, although the brush, stump, and woodchip maintained a higher level of use than the rope based on frequency of interactions and number of displacements around the enrichments.

A large amount of lying behaviour was observed on the woodchip pile in this study. Beef cattle are shown to prefer and will spend more time lying at pasture compared to a feedlot environment [25,38], and the provision of bedding materials to indoor-housed dairy cattle is a common occurrence [2]. However, there have been few studies into the provision of bedding materials to cattle at pasture, which can potentially aid in cattle comfort and cleanliness [39,40,41], and therefore reduce thermoregulatory challenges [42]. Due to higher-than-expected rainfall, it was noted that some areas of the experimental paddocks became waterlogged and muddy; however, this was not quantified. Sub-optimal conditions may prevent or reduce lying time of cattle on pasture [39,43], which has negative health impacts such as disruption to the hypothalamic-pituitary-adrenal axis [44], and increased risk of lameness [45]. Considerable individual variation in woodchip area use when provided to dairy cows at pasture has been seen, but no differences in overall lying times between those with access to the woodchip and those without were observed [23]. The relatively high level of use of the woodchip even after three weeks in the current study suggests that some aspect of the woodchip pile was viewed as favourable to the cattle, whether this was a more comfortable surface due to better drainage [39] or improved thermoregulation by keeping the cattle out of mud [46]. It is also possible that the woodchip acted as an insect repellent [47], or there was some other unknown aspect such as the height that was attractive to the cattle. This indicates the potential that exists for further research on specific bedding or structures that facilitate lying behaviour in a pasture environment, especially under sub-optimal weather conditions; however, it is likely that the woodchip would need to be regularly replaced or refreshed, as quality may decline with time.

Both the brush and stump allowed for the performance of grooming behaviours, which have been extensively studied in feedlot and dairy cattle. Over 75% of cattle in the current study interacted with the grooming brush and the stump within 11 h, which is comparable to studies in dairy cattle in which 57–79% of animals interacted with a brush within one day [8,48]. Interestingly, daily frequency of use of the brush in the current study was higher than has previously been reported for cattle housed in a feedlot [4]. Providing trees in a paddock setting to beef cattle allows for grooming that is not compensated for by other grooming behaviours, namely self- and allogrooming [12]. Similarly, providing rotating brushes to dairy calves does not reduce the amount of self-grooming and allogrooming [13]. Therefore, these enrichments facilitate the expression of grooming behaviours that would not be possible on an otherwise bare pasture, making them a potentially valuable inclusion in these environments, although further research is needed on the welfare impacts of the restriction of these behaviours.

Cattle were observed chewing woodchips and performing oral manipulations with both the rope and the stump, specifically chewing the rope and the bark and licking the root ball of the stump. Enrichments allowing for oral manipulation, specifically ropes, are provided to cattle when they may be deprived from fulfilling their natural oral behaviours of suckling and/or grazing, but mature cattle appear to interact with these ropes less; dairy: [9,10]; beef: [14,49,50]. Bark stripping is an oral stereotype seen in cattle [51,52], and is associated with a lack of foraging [16,53], some dietary deficiencies such as lack of manganese or fibre [54,55,56], or social learning [51]. Considering oral behaviours were mainly concentrated on different enrichments between cohorts (woodchip vs. stump), it is possible that this was a socially learnt behaviour. As cattle in the current study were able to graze freely, this amount and variety of oral behaviours was not expected. However, the hanging rope appeared to be the least valued enrichment item, based on use and displacements occurring around it. Rope use may have increased if the stump and woodchip were not present. Overall, it appears that some amounts of oral behaviours other than grazing, perhaps focused on exploration, may be a natural and rewarding behaviour for cattle even when housed on pasture.

It was anecdotally noted that cattle did not use enrichments when it was raining throughout the current study, instead choosing to stand without grazing as observed in other studies [57]; however, weather did not affect the likelihood of an enrichment being used, only the frequency of use given that at least one interaction occurred. It is likely that weather impacts enrichment use, for example brush use decreases under hot and humid conditions [58], whilst use of a woodchip area increases with temperature but is not affected by rainfall [23]. Our results may therefore reflect a rebound effect, in which cattle increased their use of enrichments following a brief period of rain, as weather data were based on the whole day rather than each time period. Additionally, pasture availability—whether due to seasonal or regional differences—may influence levels of enrichment use, as other behaviours such as time grazing tend to increase with decreasing pasture availability, whilst time resting decreases [15,59].

Early morning and late afternoon appeared to be the favoured times for enrichment use overall. This is in line with the activity and grazing peaks around sunrise and sunset seen in cattle housed on pasture [7,60,61], and peak brush use time from other studies [10,48,58,62]. A limitation of the current study was that observations only occurred during daylight hours, and none at night. However, previous studies examining enrichment use of dairy calves found that enrichment objects are used approximately one third less during the night than day [9], and cattle at pasture spend a large proportion of the night lying down [38].

A greater number of displacements occurred in the late afternoon in the current study, further indicating that this may be a preferred time for enrichment use, evidenced by the increased competition. However, the current study used smaller group sizes and smaller paddocks than would likely be found under commercial extensive beef grazing. Having limited access to enrichments with increasing group size may create more agonistic encounters between cattle, and potentially negatively impact welfare for lower-ranking individuals. For example, displacements per dairy cow/hour at a brush were higher than those at a feed-bunk, even though the brush was free for the majority of the day [63]. Dominant cattle have been observed to use a brush before [48] and for longer [64] than low-ranking animals at peak times. However, displacements performed and received at a brush do not differ between dominant and subordinate cows [64], and therefore it is possible that another underlying factor associated with social rank, other than competition, has an impact on the performance of rewarding behaviours and enrichment use. For example, the potential for a generally increased social stress level in subordinate cows might play a role in these relationships. Similarly, brush use is also reduced under times of stress or impaired welfare [65,66,67], highlighting that adequate physical health and nutrition should be met before benefits from enrichments may be seen. Further research is required to determine the optimal enrichment to cow ratio to minimise agonistic interactions, especially for large herds.

The cattle’s prior experience of enrichment objects was not well known, which may have impacted results. All animals were raised in commercial paddocks, and potentially had prior experience with trees and stumps but were naïve to the other objects. Repeated exposure of cattle to a novel visual object reduces the time spent exploring it and time taken to cross it [68], while repeated exposure to novel objects in a paddock decreases consistency for approach [69]. This neophobia may be reflected in the shorter latency to interact with the more familiar stump on Day 1 than the potentially less familiar woodchip pile. However, this time taken to approach may be driven by individual animals, and therefore future research may seek to determine effects of previous experiences on enrichment use. Similarly, it is unknown how enrichments may be utilised longer term in a paddock environment, with further studies across a longer period (e.g., months) required to quantify these relationships.

It was not possible to identify individual animals on each day of the study. This should be considered in future experiments, as there is considerable variation between individuals in their enrichment use [10,23,70]. Cattle also did not interact with the stump or brush for around 50% of the time they were near the object (within one body-length). This could represent a ‘queuing’ effect, which was not observed to this extent at the woodchip pile, likely due to its higher surface area, or the rope, likely due to the reduced competition over this resource. Identifying individual animals may therefore allow a closer analysis of the social aspects surrounding enrichment use, including leader–follower relationships and social learning. Additionally, specific impacts on all aspects of welfare—behavioural, physiological, and affective states—are unknown, along with any effects on production. Finally, it is not known what effect a loss of enrichment has on these cattle, which is particularly relevant in Australia due to the large variation in landscape features and the common practice of paddock rotation, along with potential loss of enrichment (either natural or artificial) when finished in a feedlot.

## 5. Conclusions

The current study investigated beef cattle preferences in and use of a variety of enrichments when housed at pasture. From these, the rope appeared to be least valued by the animals, due to reduced use and competition around it. The brush and stump, which allowed for grooming, and the woodchip, which allowed for lying, hold the most promise for the enrichment of extensively managed beef cattle, as these are highly important behaviours. It is suggested that future research should focus on determining any impacts of these enrichments on welfare and production characteristics, and how group dynamics may impact enrichment use in a larger commercial setting.

## Figures and Tables

**Table 1:** Ethogram of behaviours observed in beef cattle at pasture with access to different types of enrichments (brush, rope, stump, and woodchip pile). The time a behaviour began, the duration, and Animal ID (when visible) were recorded.

Behaviour	Description
Groom (Brush, Rope, Stump)	An animal was in physical contact (head, neck, back, or rump) with the enrichment (or post on which it is mounted for brush and rope). The animal could be either moving its body against the enrichment, or be still and leaning against it.
Oral (Rope, Stump)	Oral manipulation of the enrichment by an animal (i.e., licking, chewing).
Eat/Oral (Woodchip)	Animal consumed woodchip (to differentiate from rumination, animal’s mouth must have touched the woodchip at least once per minute).
Stand/Lie (Woodchip)	50% or more of an animal’s body (if lying) or feet (if standing) was in contact with the pile. Animal was not interacting with the woodchip in any other way (i.e., oral), but may have been ruminating.
Other (any enrichment)	Any other interaction with the relevant enrichment.
Displace (any enrichment)	An attempt to displace another animal from an enrichment, typically by headbutting.
Near (any enrichment)	Animal was standing or lying within one body-length of an enrichment, without touching or interacting with it.

**Table 2:** Hazard ratios for the latency for cattle to interact with each enrichment during the first 11 h on Day 1 of observations (total animals = 56). Significant effects are emphasized with bold font.

Enrichment	Mean Time to Interact (hh:mm)	Number Interacted	Coefficient ^1^ (±SE)	Hazard Ratio ^2^	Wald (z)	*p*
Brush	03:45	42	Reference			
Rope	03:08	34	−0.01 (±0.26)	0.99 (0.60–1.65)	−0.02	0.981
Stump	02:44	43	0.32 (±0.24)	1.38 (0.86–2.22)	1.33	0.182
Woodchip	03:49	42	−0.32 (±0.26)	0.72 (0.44–1.20)	−1.26	0.209
Rope			Reference			
Stump			0.33 (±0.27)	1.39 (0.82–2.36)	1.22	0.225
Woodchip			−0.32 (±0.27)	0.73 (0.43–1.25)	−1.15	0.249
Stump			Reference			
Woodchip			−0.64 (±0.25)	0.52 (0.32–0.86)	−2.55	**0.011**

^1^ Regression coefficient from the Cox proportional hazards model; ^2^ 95% confidence interval given in parentheses.

**Table 3:** Binomial model estimates and test statistics for the probability of an enrichment (brush, rope, stump, woodchip pile) being used by 8 groups of 7 cattle. Significant *p* values are emphasised with bold font.

Variable	Categories	Estimate (±SE)	Chi-Squared	*p*
Intercept		0.79 (±0.38)	4.42	0.036
Enrichment			12.75	**0.005**
	Brush ^a^	Reference		
	Rope ^ab^	−0.59 (±0.23)		
	Stump ^b^	−0.80 (±0.23)		
	Woodchip ^b^	−0.56 (±0.23)		
Day		−0.06 (±0.01)	18.56	**<0.001**
Time Period			50.91	**<0.001**
	Early AM ^b^	Reference		
	Late AM ^b^	−0.28 (±0.23)		
	Early PM ^b^	0.04 (±0.23)		
	Late PM ^a^	1.31 (±0.24)		
PC1		0.05 (±0.08)	0.39	0.530
PC2		−0.04 (±0.08)	0.26	0.612

^a^,^b^ Different superscript letters within variables indicate a significant difference, as determined using post hoc analyses.

**Table 4:** Conditional model estimates and test statistics for frequency of enrichments (brush, rope, stump, woodchip pile) being used by 8 groups of 7 cattle, given that an enrichment was used at least once during a time period. Significant *p* values are emphasised with bold font.

Variable	Categories	Estimate (± SE)	Chi-Squared	*p*
Intercept		2.55 (±0.07)	1506.60	<0.001
Enrichment			163.93	**<0.001**
	Brush ^a^	Reference		
	Rope ^b^	−0.79 (±0.07)		
	Stump ^a^	0.01 (±0.05)		
	Woodchip ^a^	−0.11 (±0.05)		
Day		−0.06 (±0.00)	215.91	**<0.001**
Time Period			25.34	**<0.001**
	Early AM ^a^	Reference		
	Late AM ^a^	0.02 (±0.06)		
	Early PM ^b^	−0.27 (±0.06)		
	Late PM ^a^	−0.09 (±0.05)		
PC1		−0.07 (±0.02)	14.39	**<0.001**
PC2		−0.06 (±0.02)	8.62	**0.003**

^a^,^b^ Different superscript letters within variables indicate a significant difference, as determined using post hoc analyses.

**Table 5:** Conditional model estimates and test statistics for displacements around enrichments (brush, rope, stump, woodchip pile) being used by 8 groups of 7 cattle, given that an enrichment was used at least once during a time period. Significant *p* values are emphasised with bold font.

Variable	Categories	Estimate (±SE)	Chi-Squared	*p*
Intercept		0.92 (±0.39)	5.70	0.017
Enrichment			34.87	**<0.001**
	Brush ^a^	Reference		
	Rope ^b^	−2.17 (±0.38)		
	Stump ^a^	−0.40 (±0.33)		
	Woodchip ^a^	−0.20 (±0.32)		
Day		−0.07 (±0.02)	8.80	**0.003**
Time Period			18.07	**<0.001**
	Early AM ^ab^	Reference		
	Late AM ^ab^	−0.04 (±0.39)		
	Early PM ^b^	−0.63 (±0.39)		
	Late PM ^a^	0.77 (±0.32)		
PC1		−0.17 (±0.09)	3.65	0.056
PC2		0.18 (±0.11)	2.52	0.112

^a^,^b^ Different superscript letters within variables indicate a significant difference, as determined using post hoc analyses.

## Data Availability

The data presented during this study are available on request from the corresponding author.

## Appendix A

**Table A1:** Time-period categories and the corresponding observation hours for Cohorts 1 and 2 totalling 8 groups of beef cattle, including the range of sunrise and sunset times [71].

Category	Cohort 1	Cohort 2
Early morning	07:00–10:00 (3 h)	06:30–09:30 (3 h)
Late morning	10:00–13:00 (3 h)	09:30–13:00 (3.5 h)
Early afternoon	13:00–16:00 (3 h)	13:00–16:45 (3.75 h)
Late afternoon	16:00–19:00 (3 h)	16:45–19:45 (3 h)
Sunrise	06:00–06:26	05:43–05:45
Sunset	18:57–19:13	19:33–19:48

**Table A2:** PCA loadings for daily weather variables taken over each observation day of the study.

Variable	PC1	PC2
Daily Temperature	0.600	−0.275
Daily Rain	0.491	0.415
Daily Wind	−0.395	0.713
Daily Humidity	0.493	0.493
